# School learning climate in the lens of parental involvement and school leadership: lessons for inclusiveness among public schools

**DOI:** 10.1186/s40561-020-00139-2

**Published:** 2020-09-21

**Authors:** Jason Alinsunurin

**Affiliations:** Dipartimento di Scienze Aziendali, Università degli Studi di Bologna, 40126 Bologna, Italy

**Keywords:** PISA, Parental involvement, Learning climate, School leadership, Parental coproduction, Educational inclusion

## Abstract

Prior literature has shown that school learning climate is critical in helping individual learners meet their educational objectives. In this paper, the role of parental involvement in shaping the school learning climate is explored within a multilevel and hierarchical modeling framework using data from the 2015 PISA round.

As the schools’ social and relational character, we find that reducing learning barriers is a critical challenge for school leadership. A welcoming environment for parents, as well as the effective design of effective forms of two-way communications, are positively associated with a substantial reduction in the barriers to improving teacher management’s learning climate.

We also find that public schools facing social and educational inclusiveness challenges can dramatically enhance their learning environment by activating specific parental involvement mechanisms. Similarly, principal’s leadership in framing and communicating goals and curricular development to the school is also found to be significant for inclusiveness.

However, parental involvement is also found to have potential tensions with school management. The worsening of the learning climate may arise due to pressures brought about by laws requiring parental involvement in schools. Because the learning climate is composed of a wide variety of relationships between and within schools, this work demonstrates that parental involvement is an integral part of school leadership and the school improvement process. Further research attention is encouraged to understand the tensions between teacher roles, principal leadership, and parental involvement through employing other quantitative or qualitative research designs.

## Introduction

Despite more than a hundred years of examination on school learning climate, substantial gaps and tensions persist between research findings and the practice of school leadership (Cohen, McCabe, Michelli, et al., 2009). A steady stream of literature on this field has emerged over time, which has highlighted the importance of an environment that encourages a positive learning climate among teachers, principals, and the immediate community (Thapa, Cohen, Guffey, et al., 2013). As a result, the discourse about school learning climate folds together various themes with the education management and administration literature. These have also included aspects of principals’ professionalism and school leadership (Cherkowski, 2016; Hughes & Pickeral, 2013), teachers’ job satisfaction, commitment, and well-being (Gray, Wilcox, & Nordstokke, 2017; Shoshani & Eldor, 2016) or their job burnout (Grayson & Alvarez, 2008). The dialogue and debates on learning climate have spanned the academic, policy, and professional domains—all these works convey a strong need to attain conceptual and empirical unpacking.

This work builds on this motivation to contribute to a research tradition of school learning climate, which Cohen et al. (2009) define as “*the quality and character of school life*.” Maxwell, Reynolds, Lee, et al. (2017) point it as the “*social characteristics of a school among its stakeholders”*’. As a dynamic and complex social construction, learning within schools involves a variety of actors and social structures, which lends schools its relational features. We underscore the role of parental involvement in this research inquiry, building on prior calls to understand and unpack features of this relational mechanism (Barge & Loges, 2003; Barton, Drake, Perez, et al., 2004). More explicitly, this paper asks the following research questions (RQ) through the lens of school leadership:

- RQ1: What role does parental involvement play in shaping the school learning climate?
- RQ2: Which dimensions of parental involvement matter for educational inclusiveness?

We approach to resolve the two research questions above by adopting the 2015 round of principal’s responses of the Program for International Student Assessment (PISA). The principals’ responses regarding their school’s management and leadership aspects have provided a comprehensive and in-depth view of how an organizational level construct about learning is associated with a wide variety of school characteristics. The use of the PISA dataset also allows any researcher to perform large-scale quantitative analyses with a multi-country perspective. Since the school learning climate construct(s) and parental involvement are measured at the school level, many of the findings in this research are also generalizable to national education systems. Thus, we believe that the academic and professional interest in the drivers and characterization of learning climate will be significant for the years ahead. Thapa et al. (2013) point out that research on school learning climate is an integral concern among education policy observers and scholars on managing and sustaining learning effectiveness.

The manuscript is structured as follows: Section 2 presents background literature and related theoretical and empirical work on school learning climate. Section 3 details the data and analysis, while Section 4 reports on the main results and discussion. We conclude with Section 5, present our limitations, and suggest possible directions for future work.

### Related work

Research on the theoretical conceptualization and empirical operationalization of learning climate in schools has been growing in recent years. The interest stems from the growing body of work, which underscores how conducive learning environments are associated with outcomes beyond improvements in student learning achievement. For example, a positive learning climate is associated as among the leading indicators of a well-managed school. However, the research work on the learning climate within schools is certainly not new. Still, it has a robust research tradition for more than a century (Freiberg, 2005). Professionals have long recognized the practical challenge of improving the school climate (Brookover, 1982). In its’ simplest definition, schools’ learning climate refers to the ‘quality and character of school life’ (Cohen et al., 2009), or the ‘social characteristics of a school among its stakeholders’ (Maxwell et al., 2017).

Primarily, we can characterize learning climate as a collective and shared experience, bound by interdependent social relations, group norms, shared approaches and practices, with an emphasis on learning (Cohen et al., 2009; Maxwell et al., 2017; Thapa et al., 2013). In the National School Climate Council (2007) report, Thapa et al., p. 2, (2013) cite an expanded definition:

*“School climate is based on patterns of people’s experiences of school life and reflects norms, goals, values, interpersonal relationships, teaching and learning practices, and organizational structures…A sustainable, positive school climate fosters youth development and learning necessary for a productive, contributive, and satisfying life in a democratic society; Students, families, and educators work together to develop, live, and contribute to a shared school vision. Educators model and nurture an attitude that emphasizes the benefits and satisfaction from learning. Each person contributes to the operations of the school as well as the care of the physical environment*.” (Thapa et al., 2013)

Despite the new challenges in its definition, the learning climate is known to link to many learning outcomes, most of which are related to academic performance. The latest research shows that a positive learning climate figures in students’ resilience (Domitrovich, Durlak, Staley, et al., 2017), students’ improved health behaviors and choices (Michael, Merlo, Basch, et al., 2015), the reduction of socio-economic and racial gaps (Berkowitz, Moore, Astor, et al., 2017; Sanders, Durbin, Anderson, et al., 2018; Voight, Hanson, O’Malley, et al., 2015), the reduction in alcohol and marijuana use (Cornell & Huang, 2016), and considerable substantial reduction in peer bullying, aggression, teasing, and general victimization (Cornell, Shukla, & Konold, 2015; Gage, Prykanowski, & Larson, 2014; Konishi, Miyazaki, Hymel, et al., 2017; Konold, Cornell, Huang, et al., 2014; Wang, Vaillancourt, Brittain, et al., 2014). It is also known to induce a student’s potential political participation (Castillo, Miranda, Bonhomme, et al., 2015), prosocial behavior (Luengo Kanacri, Eisenberg, Thartori, et al., 2017; Luengo Kanacri, Pastorelli, Zuffianò, et al., 2014), and the likelihood not to drop out of school (Jia, Konold, & Cornell, 2016).

In most of these works, researchers have recognized the multidimensional and the multi-domain construct of learning climate. Authors such as Wang and Degol (2016) have mentioned that these dimensions of the school climate fall into four main domains: academic, community, safety, and institutional environments. This finding complements an earlier review performed by Thapa et al. (2013), which included teaching and learning, school improvement processes, and school relationships. Altogether, these dimensions capture almost every aspect of the school environment for learning, reinforcing drivers of students’ cognitive, behavioral, and psychological development (Wang & Degol, 2016). However, recent contributions to the literature focus on case studies of schools’ learning climate within highly specific contexts. There are still few studies which look at the generalizable aspect of school learning climate in a multi-country perspective.

#### Unpacking the mechanism of school climate improvement process

School climate is essentially a dynamic and complex social construction: there are a variety of actors such as school principals, counselors, teachers, and parents that characterize its relational features. Given the present literature on the various outcomes which are reinforced with a positive or improved learning climate, attractive policy and practice questions certainly arise. These include which dimensions of school management and contextual characteristics directly relate to the learning climate. Sebastian, Allensworth, and Stevens (2014) and Sebastian and Allensworth (2012) and building on Bryk (2010), they provide a conceptualization of such a mechanism. School leadership works through three mediating processes to influence teaching and learning in schools. These include school staff professional capacity, the learning climate, and the parent-community ties. The interplay of these processes within a school’s context directly influences student learning through classroom instruction and indirectly through the school context. Professional capacity captures teachers’ professional qualifications, the schools’ quality assurance coordination programs and the general professional community.

Hallinger, Bickman, and Davis (1996) found no direct impact of the principal’s instructional leadership on student achievement. Still, they find substantial evidence to point out that principals can have an indirect effect on the school learning climate. This relationship highlights the role of school leaders’ role in overseeing school effectiveness. This conceptualization implies that in studying the school learning climate, one cannot isolate it with variables such as the principal’s leadership, teachers’ roles in school management, and the school’s institutional setup.

The school’s institutional or organizational features also influence the school’s learning climate (Wang & Degol, 2016). Across the world, the majority of schools are public. They receive government financing, and they are subject to public accountability rules. In this sense, teachers, principals, and other school staff are, by and large, public sector employees accountable to the demands and expectations of their profession and the government. Therefore, research on schools’ learning climates also extends beyond the interest of educational management and leadership scholars, but also to scholars of public accountability, organizational behavior, and public personnel management.

#### Parental involvement and school learning climate improvement

In this section, we revisit the literature on school improvement and parental improvement. For a long time, parental involvement is known to be among the predictors of students’ academic achievement (Barge & Loges, 2003). It comes as no surprise why parental involvement in education settings remains one of the most critical areas of educational policy research. In varying levels of attention, parental involvement is one of the vital pillars of comprehensive education reform programs across the world. In a critical study in 2004, Barton et al. (2004) have characterized parental involvement as virtually a co-productive and interactive process by parents with schools. It has been described as a “*dynamic, interactive process in which parents draw on multiple experiences and resources to define their interactions with schools and among school actors*” (p. 3; (Barton et al., 2004)). The authors also discuss that while in general, most of the literature focuses on the “what” part of parental involvement, but little attention has been paid to its dimensions such as “its why’s and it’s how’s.”

In particular, most of the research attention on parental involvement has mostly focused on the “visibility of parents” in schools as a determinant of academic achievement (Kim, 2009). On the other hand, there is also a scarcity of research focusing on engaging parents as equal partners and decision-makers within education communities (Barton et al., 2004). How certain typologies of parental involvement relates to a specific domain or pillar of the school’s learning climate is less known. This quite surprising as the literature on the school climate has underscored the importance of interpersonal relationships between school personnel and other school actors (Sebastian & Allensworth, 2012). Previous studies have also called for coordinated action results in an improvement in outcomes measured at the student-level outcomes, much expectedly less so for organizational-level indicators such as the learning climate. The empirical literature remains scant when it comes to an understanding of the mechanisms through which specific or contextual varieties of parental involvement relate to schools’ learning climate.

Parental involvement in schools is a vital component of the design of intervention programs such as anti-bullying and victimization (Georgiou, 2008). Also to address issues of mental health and treatment of OCD among teenagers (Derisley, Libby, Clark, et al., 2005); the design of programs to share direct and indirect responsibilities in diabetes management among youth (Young, Lord, Patel, et al., 2014); as well as the long term involvement by parents to enhance diabetes management efficacy among the adolescents (King, Berg, Butner, et al., 2014). Health outcomes of children may also improve by engaging parents, such as in case of early intervention programs with kids facing hearing loss (Ingber & Dromi, 2009) and in reducing the likelihood of developing smoking addiction (Kestilä, Koskinen, Martelin, et al., 2006). These results are remarkable for youth and adolescent outcomes; the literature still faces the gap in how parental involvement may also relate with a school (or organizational) level indicator, such as the learning climate.

Moreover, attitudes towards parental involvement within schools vary. Empirical studies such as Addi-Raccah and Ainhoren (2009) discussed Israel, where teacher attitudes were mostly negative and resistant in schools where parents are empowered. Even in cases where teachers favor involvement, teachers felt they are susceptible to expanding the influence of parents who “scrutinize their work and encroach their professional domains” (Addi-Raccah & Arviv-Elyashiv, 2008). In a similar instance, Bæck (2010) also found that in Norway’s case, parents can potentially undermine teachers’ autonomy in the classroom. Teachers sought restricting parental involvement, especially among well-educated parents. The teaching staff is known to emphasize their own professional identity in the classrooms. Teacher reports of parental responsibility to influence student outcomes are more robust than that of parental statements implying that “stereotyping” of parents by teachers can affect academic results (Bakker, Denessen, & Brus-Laeven, 2007).

## Data and methods

### Data used

For this study, we used principals’ responses from the 2015 round of the Program for International Student Assessment (PISA) downloaded from https://www.oecd.org/pisa/data/2015database/. As the broadest education assessment program in the world, it has the most extensive and generalizable multi-country survey on academic achievement collected alongside parental involvement. The main unit of observation is the principal’s responses from more than 16,000 schools across all 62 PISA participating countries.

### Use of latent indicators

To understand the underlying factor structure of some of the latent factors of interest, we follow a two-step approach described by Anderson and Gerbing (1988). First, we conducted preliminary tests such as exploratory factor analysis (EFA) and confirmatory factor analyses (CFA). It is done by randomly dividing the dataset into two parts and then performing an EFA in the first part to show which variables can be grouped (the “training set”). We subsequently examined whether the same structure applies to the other half by performing a CFA. This approach will be advantageous in our subsequent regression analyses to reduce the possibility that variables comprising a latent structure are determined by chance. And then, finally, to generate the composite indicator for the latent variable, we followed this by performing confirmatory factor analyses for the full sample. We also conducted Bartlett’s test and computed for the KMO measure. We begin the empirical section by assessing how principals and school heads perceive parental involvement.

### Estimation strategy

As a cross country study of schools in a single period, we have a hierarchical data structure: the unit of observations (schools) are nested within countries’ education systems. We specify a two-level model where it allows us to simultaneously investigate the relationship of the school learning climate and several variables measured at the school level, as well as having the ability to compare measurements between levels, i.e., variation between countries. The model takes the simple form,

$$ {\mathrm{Y}}_{\mathrm{ij}}={\upbeta}_{\mathrm{oj}}+{\upbeta}_{1\mathrm{j}}\left({\mathbf{PI}}_{\mathrm{ij}}\right)+{\upbeta}_{\mathrm{ij}}{\mathrm{X}}_{\mathrm{ij}}+{\upgamma}_{\mathrm{ij}} $$

Where *Y*_*ij*_ is a measure of a learning climate in school *i* nested within a country *j*. The vector ***X***_*ij*_ contains the control variables discussed above; **PI** are indicators of parental involvement observed at the level of the school, the coefficient *β*_*oj*_ is the expected level of learning climate when all other explanatory variables are equal to zero. *β*_1*j*_ and *β*_*ij*_ are the respective beta coefficients. and *γ*_*ij*_ is a random error associated with the level of schools nested within the country.

### The dependent variable

#### The school’s learning climate

There are several ways to assess the learning climate in schools and these assessments depend on the scope of the respondent’s perspective. As we are concerned with an organizational level feature, we consider principals’ assessments to be a potential vantage point to assess such. In the PISA survey, principals were asked ten questions, “*In your school, to what extent is the learning of students hindered by the following phenomena*?” and may respond with any of the four choices in an ordinal scale, i.e., *1-Not at all; 2- Very little; 3- To some extent; 4- A lot*. Table 4 in the Additional file 1 shows us the summary statistics of these ten variables, where two potential constructs seem to emerge. The first measure captures *the extent that student-related issues hinder learning*. In contrast, the second measure is confirmed to be the *scope by which learning is hindered by teacher management issues*, as perceived by the principal. We clarify that our approach in measuring the learning climate takes only the school principal’s perspective; this, we believe, that school leadership endows us an excellent position to assess an organizational-level outcome.

We performed Anderson and Gerbing’s approach to testing the compositeness of our outcome variables of interest. Initially, the ten items are expected to arrive with two measures of the school level’s learning climate neatly. However, the second measure is a statistically more robust and consistent measure of the learning climate. This is in line with the principle that the dependent variable(s)’ operationalization must be consistent across all responses.

The first measure, *the extent that student-related issues hinder learning,* has shown that the underlying construct is not always consistently viewed as homogeneous in our broad sample of countries. It has an RMSEA of 0.230 as compared to the RMSEA of the second measure at 0.052. As constructed indices, we concentrate our analysis of the learning climate on the second factor or the extent that teacher-related issues which hinder the learning climate. This composite index is a simple average of the five items on teacher management/behaviors. The subdimensions consider five dimensions:

- Teachers’ ability to meet the needs of students,
- Teacher absenteeism,
- Staff resistance,
- Teachers being too strict, and
- Teachers’ preparedness.

The average inter-item correlation for these items is 0.4685, and the Cronbach’s α is 0.82. These measures and their statistical properties are fully reported in the Additional file 1, Table 1.

### Explanatory variable/s and controls

#### Parental involvement

These are parental involvement dimensions as perceived by the school principal, found in the SC063 of the principal’s survey. Principals answer statements about how much parental involvement apply in their school. In contrast to the student-parent questionnaire, the responses on this module of the survey are dichotomous (Yes/No).^1^ Preliminary correlation matrices of these six items show a very low to low correlation (min 0.08, max 0.26). Thus, we do not expect this to cause multicollinearity. Still, we have exercised further caution by testing for the variance inflation factor (VIF) contributions across all of our regression analyses. The VIF contributions have remained low throughout.

#### Principal leadership

The principal leadership module contains 13 items in section SC009. We followed the similar technique of randomly dividing the dataset into two parts and testing whether the EFA is congruent with the CFA. The exploratory factor analysis on the training dataset yielded two possible factors. Upon conducting the CFA, the groupings did not indicate a good fit based on the two-factor model of principal leadership. The RMSEA showed 0.106, and the CFI and TLI measures are below 0.90. These results likely indicate unfitness to drastically reduce the number of dimensions from 13 to just two. Furthermore, while reducing the factors into only two may theoretically lower the likelihood of multicollinearity, but the interpretation becomes a practical challenge. This finding includes the separability of which specific dimensions of principal leadership influence school outcomes.

Fortunately, for this round of the PISA, OECD has pre-determined the item parameters to capture distinct types of principal leadership adequately. These are based on the prior rounds of PISA, a four-factor model of school leadership. These four factors were computed and derived by the OECD based on the item-response theory (IRT) scaling. These leadership dimensions included curriculum development (LEADCOM), instructional leadership (LEADINST), professional development (LEADPD), and teachers’ participation (LEADTCH). These combinations of items are all tested in the CFA four-factor framework, which resulted in a reasonably well-fitted model. For the regression analyses, we integrated these dimensions in our estimates. The full summary statistics of these variables are shown in Additional file 1 Table 2.

### Other control variables

#### School autonomy

School autonomy Is assessed in the survey through 12 items. Principals were asked who has considerable authority for hiring teachers, setting salaries, formulating the budget, managing resources, and designing the curriculum, among others. Four derived indicators are taken from these 12 items, RESPCUR, the responsibility of the school staff with issues relating to curriculum and assessment, and RESPRES, an index of relative responsibility of staff in managing school resources. Both indices were standardized with a mean of 0 and a standard deviation of 1. The overall index school autonomy, SCHAUT, was computed as the percentage of the items for which school staff, teachers, or the school governing board have the most responsibility. These are thoroughly discussed in Annex A of the PISA Technical Background. On the other hand, TEACHPART, teacher participation is a simple sum of the number of items where teachers have the most authority. We also performed analyses of distinguishing internal and external evaluation practices of schools, defined below.

#### Student assessment

We have included and constructed controls for the use of student assessment and evaluation, STANTEST1 (standardized tests for information for decision-making purposes), and STANTEST2 (standardized tests for practices of comparison), following the stringent EFA-CFA checks.^2^

#### Other controls

We included the following controls for our analyses: EDUSHORT, to capture the shortage of educational materials. STAFFSHORT, an index to measuring the lack of instructional staff. To include teacher quality, we include PROATCE, an index measuring the proportion of teachers in a school who are fully certified with teaching qualifications. Moreover, we distinguish between different types of schools, denoted by SCHLTYPE. PISA reports whether a school is privately independent, private, but government-dependent or a public school. The student-teacher ratio is reported through the variable STRATIO (Table 1).

**Table 1:** Summary statistics of the item components of parental involvement in the principal questionnaire^a^

CODE	Full sample					Private independent schools			Private government-dependent schools			Public schools		
	Obs	Mean	Std. Dev.	Min	Max	Obs	Mean	Std. Dev.	Obs	Mean	Std. Dev.	Obs	Mean	Std. Dev.
SC063Q02NA	16,096	0.97	0.17	0	1	1362	0.98	0.14	1479	0.99	0.12	12,279	0.97	0.16
SC063Q03NA	16,089	0.92	0.27	0	1	1362	0.95	0.22	1482	0.95	0.22	12,268	0.90	0.29
SC063Q04NA	16,044	0.78	0.41	0	1	1352	0.63	0.48	1480	0.71	0.45	12,239	0.80	0.40
SC063Q06NA	16,046	0.90	0.30	0	1	1358	0.93	0.25	1479	0.91	0.29	12,239	0.90	0.30
SC063Q07NA	16,011	0.76	0.43	0	1	1356	0.75	0.43	1472	0.78	0.42	12,222	0.76	0.43
SC063Q09NA	16,000	0.70	0.46	0	1	1356	0.54	0.50	1476	0.55	0.50	12,216	0.74	0.44

Note: Definition of items (taken from the PISA 2015 questionnaire) are supplied below. Note that the items are recoded in a such a way that 0 implies NO, and 1 imply YES. The means automatically generalize the proportion of involvement in percentage terms

^a^Full source: http://www.oecd.org/pisa/data/CY6_QST_MS_SCQ_CBA_Final.pdf

Variable codes

SC063Q02NA Our school provides a welcoming and accepting atmosphere for parents to get involved

SC063Q03NA Our school designs effective forms of school-to-home and home-to-school communications about school programs and students' progress

SC063Q04NA Our school includes parents in school decisions

SC063Q06NA Our school provides information and ideas to families about how to help students at home with homework and other curriculum-related activities, decisions, and planning

SC063Q07NA Our school identifies and integrates resources and services from the community to strengthen school programs, family practices, and student learning and development

SC063Q09NA There is federal, state, or district legislation on including parents in school activities

## Results and findings

Before estimating the HLM models, we tested several econometric specifications with ordinary least squares (OLS) to check several modeling parameters. We checked the stability of signs, the magnitude of the beta coefficients, and the overall model fit. Owing to the nested nature of schools within countries, we computed for the intraclass correlation coefficients (ICC) after the HLM estimates. This technique enabled us to estimate the proportion of variation in the school climate explained by school clustering. The ICC values across all the HLM models ranged only from 0.11 to 0.18. The values indicate that principals’ beliefs about the learning climate within their schools are not so much different from the observations of principals in other countries. Because a low ICC implies little variability between clusters, a more straightforward random effects estimation will suffice as an estimation strategy.

For purposes of empirical and presentational clarity, only the estimates from HLM specifications are reported. All calculations were computed with robust standard errors; VIF tests yielded values between 2.2 and 2.8 across all specifications. This value indicates a low likelihood of a high correlation among our chosen predictor variables. The adjusted R-squared of our random-effects models also shows a reasonable explanatory variation of the learning climate, which can be accounted for by the explanatory variables ranged from 11.3% to 23.9%. The F-tests also indicated a good fit of the model across all specifications, with *p*-values below 0.01. We find similar and consistent results among our random-effects models and HLM estimates. Still, HLM models are used to draw the analyses and discussion. This approach is more sensible and illustrates the nested nature of schools within education systems.

The HLM model extensions drew estimates where observations report full data availability. Non-responses in some of the individual sections of the questionnaires in some modules are not available in other countries. This situation led to a slight decrease in the number of school-principal reports from more than 15,000 total schools to about 10,900 in the most restrictive sample included in the full regression analyses. The number of countries included in each pooled regression is shown below each regression table.

### RQ1: the role of parental involvement in improving the learning climate

The initial HLM results are in Table 2 below. Our preliminary analyses show that public school principals face the worst barriers in improving the learning climate attributable to teacher behavior/management issues. It is essential to determine which among parental involvement dimensions are associated with the reduction in barriers in improving the learning climate. We have formulated various specifications where we include other controls one by one, pooling all the observations where we have complete information. This way, we get to see sensitivity and magnitude stability among the coefficients of parental involvement dimensions. We find the signs are generally consistent and show no severe change in terms of magnitude. Full specification models were also replicated for subsets of public and private schools.

**Table 2:** Hierarchical Linear Modeling (HLM) estimates on the extent of how learning is hindered due to teacher behavior/management issues

Variables	Pooled 1	Pooled 2	Pooled 3	Pooled 4	Pooled 5	Public schools only	Private schools only
1.SC063Q02NA	-0.158*** (0.0498)	- 0.199*** (0.0549)	- 0.195*** (0.0550)	-0.224*** (0.0603)	- 0.224*** (0.0605)	- 0.231*** (0.0715)	- 0.165 (0.107)
1.SC063Q03NA	-0.197*** (0.0254)	- 0.176*** (0.0277)	- 0.154*** (0.0304)	-0.180*** (0.0380)	- 0.182*** (0.0380)	- 0.204*** (0.0398)	- 0.0347 (0.0854)
1.SC063Q04NA	-0.0132 (0.0168)	-0.0224 (0.0150)	- 0.0229 (0.0153)	-0.00646 (0.0187)	- 0.00486 (0.0192)	-0.0329 (0.0229)	0.0478 (0.0480)
1.SC063Q06NA	-0.106*** (0.0245)	- 0.101*** (0.0283)	- 0.0906*** (0.0294)	- 0.0886** (0.0356)	- 0.0872** (0.0356)	-0.0988*** (0.0372)	0.00243 (0.0806)
1.SC063Q07NA	-0.0730*** (0.0141)	- 0.0819*** (0.0158)	-0.0728*** (0.0169)	- 0.0773*** (0.0183)	-0.0767*** (0.0182)	- 0.0621*** (0.0198)	-0.0942*** (0.0333)
1.SC063Q09NA	0.0555*** (0.0194)	0.0174 (0.0181)	0.0239 (0.0184)	0.0211 (0.0201)	0.0195 (0.0202)	0.0241 (0.0223)	-0.0175 (0.0396)
LEADCOM			-0.0624*** (0.0162)	-0.0681*** (0.0161)	- 0.0680*** (0.0161)	-0.0491*** (0.0145)	- 0.107** (0.0495)
LEADINST			0.00974 (0.0128)	0.0120 (0.0128)	0.0117 (0.0129)	0.0100 (0.0137)	-0.0114 (0.0251)
LEADPD			-0.00133 (0.0111)	-0.00393 (0.0120)	- 0.00402 (0.0120)	-0.00917 (0.0117)	0.0140 (0.0193)
LEADTCH			0.00249 (0.0121)	0.00638 (0.0110)	0.00658 (0.0109)	-0.00401 (0.0120)	0.0430 (0.0317)
XSTANTEST1				0.0379 (0.0282)	0.0387 (0.0281)	0.0455 (0.0304)	0.0145 (0.0478)
XSTANTEST2				-0.0728*** (0.0230)	-0.0725*** (0.0230)	-0.0640** (0.0264)	-0.120*** (0.0394)
RESPCUR					-0.0139 (0.0102)	-0.0100 (0.0123)	-0.0351* (0.0200)
RESPRES					0.00562 (0.0146)	0.00690 (0.0112)	0.0136 (0.0308)
SCHAUT		-0.0162 (0.0595)	-0.0164 (0.0623)	-0.0214 (0.0643)	-0.0153 (0.0705)	0.0693 (0.0663)	-0.137 (0.188)
TEACHPART		0.00424 (0.00481)	0.00395 (0.00457)	0.00305 (0.00458)	0.00385 (0.00481)	-0.00631 (0.00545)	0.0186* (0.00965)
EDUSHORT		0.111*** (0.0121)	0.111*** (0.0123)	0.107*** (0.0139)	0.107*** (0.0139)	0.100*** (0.0155)	0.121*** (0.0213)
PROATCE		-0.0313 (0.0317)	-0.0163 (0.0325)	0.00128 (0.0337)	0.000525 (0.0337)	-0.0135 (0.0401)	0.0808 (0.0546)
2.SCHLTYPE		0.0569 (0.0490)	0.0407 (0.0512)	0.0487 (0.0460)	0.0493 (0.0441)		0.0553 (0.0426)
3.SCHLTYPE		0.177*** (0.0433)	0.157*** (0.0453)	0.148*** (0.0429)	0.150*** (0.0449)		
CLSIZE		0.00412*** (0.00133)	0.00451*** (0.00125)	0.00519*** (0.00144)	0.00529*** (0.00142)	0.00594*** (0.00134)	0.00197 (0.00394)
STRATIO		0.00234** (0.00115)	0.00242** (0.00114)	0.00264** (0.00128)	0.00264** (0.00126)	0.00197 (0.00135)	0.00141 (0.00203)
Constant	2.325*** (0.0703)	2.112*** (0.106)	2.070*** (0.102)	2.099*** (0.110)	2.085*** (0.108)	2.264*** (0.102)	1.906*** (0.213)
Observations	15,708	12,572	12,109	10,516	10,505	8520	1985
Number of countries	69	64	64	63	63	63	63

Robust standard errors in parentheses

*** *p* < 0.01, ** *p* < 0.05, * *p* < 0.1

To answer the first research question, we find that four out of the six dimensions of parental involvement reduce the hindrances to improving the learning climate with various size effects. First, the analysis shows providing a welcoming and accepting environment for parental participation is significantly and positively associated with the improvement of the learning climate. The gain ranges from 0.16 to 0.22 points, and the effect is consistent and robust across school types. In the PISA school survey, public schools, on the average, have worse indicators of learning climates than private schools. Thus, a friendly environment for parents should be an essential point for school improvement among public school principals.

**Fig. 1:**
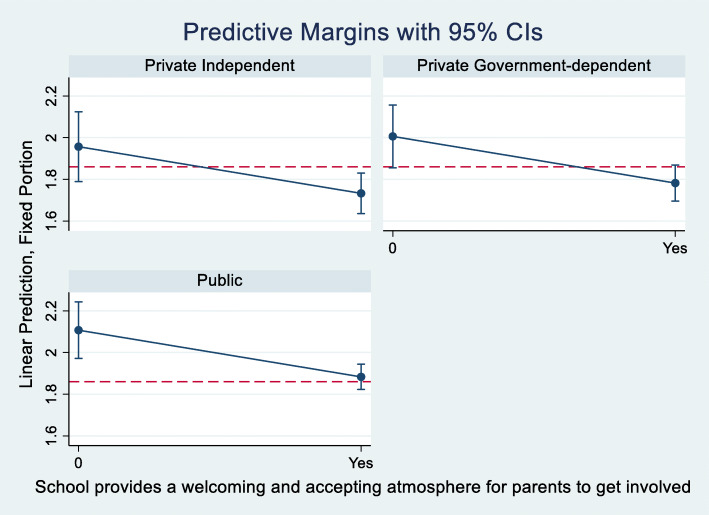
Improvements in reducing learning climate barriers associated with a welcoming and accepting atmosphere for parental involvement. Graphic computed using pooled model #5. The red dashed line denotes the average score of learning climate associated with teacher behavior, 1.86

**Fig. 2:**
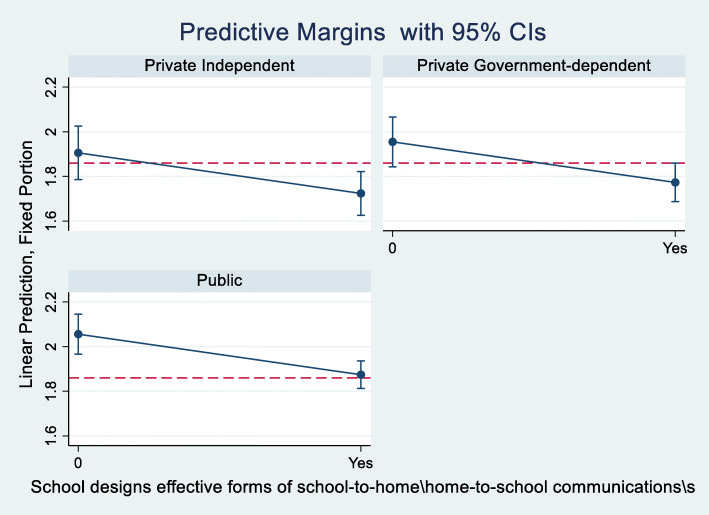
Improvements in reducing learning climate barriers associated with designing effective forms of school-to-home and home-to-school communications. Graphic computed using pooled model #5. The red dashed line denotes the average score of learning climate associated with teacher behavior, 1.86

We also find that designing effective communication channels about school programs and students’ progress (SC063Q03NA) is associated with improvements in learning climate. The improvements range from -0.15 to -0.20- point reductions in barriers to the learning climate associated with teacher behavior/management issues. Predictive margins computed from the pooled model #5, are shown in Figs. 1 and 2 for illustration, clearly underscoring the importance of communication channels between schools, teachers, and parents.

Two involvement dimensions are associated with the reduction in the barriers to the learning climate. Schools’ provision of information and ideas about how families can help with the child’s homework and other curriculum-activities (SC063Q06NA) and schools’ integration of resources and services from the community (SC063Q07NA) are statistically significant. While the magnitude is not as pronounced as SC063Q02NA and SC063Q03NA, this is still a big lesson for schools. These results point that parents’ community and home-based educational coproduction activities are inseparably linked with that of principals’ managerial realm. Moreover, this appeals particularly to teacher behavior and management.

It is also essential to point the role of other parental involvement indicators in the rest of the models. Including parents in school decisions (SC063Q04NA) is not statistically significant across all HLM models. The role of national/federal/local legislation on parental involvement appears limited. Despite other parental involvement indicators being significant among the pooled models (specifications 1 to 5, as well as public schools), only one parental indicator is significant for private schools, the school resource integration to strengthen school programs. The rest of parental involvement indicators matter mostly for public schools.

Among the school leadership controls, only LEADCOM (the principal’s leadership in framing and communicating school goals and curricular development) relates to a reduction in the hindrances in the learning climate. However, its magnitude is lower than those of parental involvement. This finding is evidence of gaps between private and public schools. The adoption of standardized tests for practices of comparison (STANTEST2) is associated with lower barriers in the learning climate among private schools, which is twice as much as public schools. Large class sizes (CLSIZE) in private schools do not significantly relate to the learning climate hindrance. In contrast, it is a significant barrier among public schools. Prior works cite teachers’ challenges in managing large class sizes and reduced teacher-pupil interactions (Clinton & Hattie, 2013; Hattie, 2005; Pedder, 2006).

### RQ2: the role of parental involvement in fostering socially inclusive learning environments in public schools

In this section, we answer RQ2: to identify the domains of parental involvement that matters for educational inclusiveness. The analyses were extended within a subset of public schools where the challenges in managing the learning climate are likely to be more noticeable.

The PISA survey asked principals to estimate the percentage of students in their school (1) whose heritage language is different from the test language, (2) who have special needs (SEN), and (3) who come from economically disadvantaged homes. Principals’ and teachers’ roles within these schools facilitate schools’ capacity to ensure that students face no risk of being excluded. Given that we measure learning climate in terms of its association with teacher behavior/management issues, this research underscores and emphasizes the relational character of learning climate within communities.

On the practical side, there are several reasons why improving school climate remains a challenge based on educational inclusion perspectives. Many students face language barriers that have contributed to difficulties in adjusting to the school environment. These are in cases where a different language is spoken at home (Verwiebe & Riederer, 2013). In terms of academic performance, prior studies have documented gaps between children of natives and immigrants (Zinovyeva, Felgueroso, & Vazquez, 2014), as well as their inter-generational differences (Schleicher, 2006).

Moreover, the inclusion of students with SENs in educational evaluation involving PISA has always been challenging. It has mainly been a consequence of the limited sample representation in the prior survey waves, which is attributable to the strict sampling criteria and the national laws requiring the inclusion of SEN students (LeRoy, Samuel, Deluca, et al., 2019). The school survey only asks principals of their estimates of SEN enrollment shares. The OECD even further mentions that the methodology needs further refinement^3^ to ensure the representation of students with SENs.

Furthermore, due to the heterogeneity in education systems and policies, not all schools have the same level of representation for students with SENs. There are existing guidelines on how SEN students may participate based on the OECD’s exclusion criteria (LeRoy et al., 2019). Yet, empirical studies examining equity and educational inclusion must be conscious of such limitations. PISA’s technical and user guides discuss the assessment’s inclusion and exclusion criteria, providing analyses on PISA sufficient information about the inclusion of SENs across a broad set of schools.

Six HLM regression estimates are reported for each subset of public schools across PISA sample countries whose share of the student enrollment exceeds 20% and 50% in the categories. These subsets are various student shares of student populations with SENs, using different language at home, and students with from socio-economically disadvantaged backgrounds. The ICC values have remained below 0.30 in most of the specifications. This finding indicates that principals’ perceptions in these subsets of public schools do not perceive their schools to be substantially very different from principals’ observations in other countries. Significantly limiting the analyses to the subset of these schools emphasizes schools’ learning climate challenges under these circumstances.

The overarching message we find within this section: the role in which parental involvement plays cannot be discounted nor understated by public school principals (see Table 3 below). First, substantial improvement persists in reducing barriers to the learning climate linked to teacher behavior and management when public schools provide a welcoming and accepting atmosphere for parents to get involved, β_1.SC063Q02NA_ ≈ - 0.217 to - 0.401). The improvement is remarkable among public schools whose share of the student population with SENs is > 50% (column 5). This also applies as well as for schools with > 50% socio-economically disadvantaged students (column 7), as well as schools with > 50% of students whose heritage language is different from the language of instruction (column 3). Moreover, parental involvement in the form of school-to-home and/or home-to-school communications is also significant (β_1.SC063Q03NA_ ≈ - 0.147 to - 0.242). A relevant, actionable course of action for school leadership is to ensure that schools retain an inclusive social character, with clear and accessible communication lines between principals, teachers, and parents.

**Table 3:** HLM estimates on subsets of schools grouped by the proportion of students coming from various potentially vulnerable groups

VARIABLES	Public with the proportion of different language > 20%	Public with the proportion of different language > 50%	Public with students with special needs > 20%	Public with students with special needs > 50%	Public with students from socio-economically disadvantaged homes > 20%	Public with students from socio-economically disadvantaged homes > 50%
1.SC063Q02NA	- 0.242*** (0.0795)	- 0.271** (0.114)	- 0.265 (0.180)	- 0.401*** (0.146)	- 0.217*** (0.0528)	- 0.275*** (0.0988)
1.SC063Q03NA	-0.170*** (0.0461)	- 0.179*** (0.0478)	- 0.242*** (0.0596)	- 0.124* (0.0696)	- 0.147*** (0.0388)	-0.182*** (0.0519)
1.SC063Q04NA	-0.0580* (0.0337)	- 0.0561 (0.0407)	-0.0259 (0.0491)	- 0.0308 (0.0529)	-0.0851*** (0.0265)	- 0.0883** (0.0387)
1.SC063Q06NA	- 0.0648 (0.0475)	- 0.0852 (0.0575)	- 0.181** (0.0846)	- 0.116 (0.0717)	- 0.0749** (0.0327)	- 0.0846 (0.0616)
1.SC063Q07NA	-0.0538* (0.0310)	- 0.0543 (0.0350)	- 0.0967*** (0.0323)	-0.0811* (0.0449)	- 0.0574*** (0.0216)	-0.0606* (0.0312)
1.SC063Q09NA	0.0772*** (0.0293)	0.0789*** (0.0253)	0.0517 (0.0440)	0.0654 (0.0490)	0.0652** (0.0294)	0.0627* (0.0362)
LEADCOM	-0.0636*** (0.0221)	-0.0489* (0.0274)	-0.0467 (0.0290)	-0.0602 (0.0396)	- 0.0424** (0.0207)	-0.0545* (0.0299)
LEADINST	-0.00163 (0.0243)	- 0.0202 (0.0269)	0.0114 (0.0276)	0.000366 (0.0335)	0.00733 (0.0160)	0.0217 (0.0240)
LEADPD	-0.0253 (0.0199)	-0.0187 (0.0211)	-0.0519* (0.0267)	- 0.0253 (0.0313)	-0.0133 (0.0172)	- 0.00109 (0.0226)
LEADTCH	0.00409 (0.0171)	0.0189 (0.0187)	-0.00203 (0.0243)	-0.0213 (0.0357)	- 0.0101 (0.0156)	-0.00430 (0.0188)
XSTANTEST1	0.0889 (0.0568)	0.119* (0.0628)	0.0911 (0.0587)	0.108 (0.0700)	0.0102 (0.0391)	0.0378 (0.0572)
XSTANTEST2	-0.0510 (0.0491)	-0.0832* (0.0466)	-0.0303 (0.0697)	0.0478 (0.0986)	-0.0557 (0.0354)	-0.0193 (0.0435)
RESPCUR	-0.0321** (0.0160)	-0.0279* (0.0163)	- 0.0123 (0.0243)	0.0333 (0.0323)	- 0.0249 (0.0188)	-0.0177 (0.0259)
RESPRES	0.0201 (0.0211)	0.0200 (0.0172)	0.0188 (0.0197)	0.0429 (0.0265)	0.0119 (0.0147)	0.0352 (0.0247)
SCHAUT	0.135 (0.117)	0.219* (0.131)	-0.0429 (0.163)	-0.0666 (0.207)	0.111 (0.0917)	-0.0783 (0.121)
TEACHPART	-0.00623 (0.00877)	-0.00744 (0.00863)	- 0.000916 (0.0135)	0.00717 (0.0165)	- 0.00851 (0.00754)	-0.00885 (0.00984)
EDUSHORT	0.0967*** (0.0204)	0.0963*** (0.0216)	0.119*** (0.0270)	0.127*** (0.0382)	0.0745*** (0.0151)	0.0665*** (0.0179)
PROATCE	-0.0441 (0.0431)	-0.0368 (0.0524)	-0.0102 (0.0731)	-0.0660 (0.0930)	- 0.0157 (0.0523)	-0.0509 (0.0681)
CLSIZE	0.00640*** (0.00153)	0.00608*** (0.00156)	0.00759*** (0.00178)	0.00345 (0.00210)	0.00550*** (0.00145)	0.00676*** (0.00173)
STRATIO	0.00115 (0.00178)	0.000798 (0.00217)	0.00264 (0.00272)	0.00623* (0.00337)	0.00225 (0.00153)	0.00173 (0.00187)
Constant	2.142*** (0.126)	2.137*** (0.156)	2.383*** (0.169)	2.333*** (0.214)	2.211*** (0.132)	2.380*** (0.183)
Observations	3338	2629	2052	1130	4816	2348
Number of groups	62	62	61	60	63	62

Robust standard errors in parentheses

*** *p* < 0.01, ** *p* < 0.05, * *p* < 0.1

On the other hand, we find legislation on including parents in school activities is associated with a slight worsening of the learning climate. This observation applies to public schools with more than 20% of students whose heritage language is different from the language of instruction (β_1.SC063Q09NA_ ≈ 0.0772 to 0.0789, columns 2 and 3). The result indicates and confirms the tensions and conflicts on how school leadership manages issues of cultural diversity (Herrity & Glasman, 2010). This friction has also been frequently made complex due to communication problems and stereotyping, which Grobler, Moloi, Loock, et al. (2006) have previously indicated.

Several aspects of the principal’s leadership dimension also arise. We find the principal’s leadership in framing and communicating school goals and curricular development (LEADCOM) to be significantly associated with a reduction in the extent that learning climate is hindered (columns 2 and 6). Principals’ promotion of instructional improvements and professional development among teachers (LEADPD) is also significantly associated with a decrease in the learning hindrances among schools with more than 20% of students with SEN’s (column 4).

Coincidentally, in this level, schools providing a welcoming and accepting atmosphere for parents to get involved (SC063Q02NA) is associated with a substantial reduction in the barriers to learning among public schools with more than 50% of students with SENs. The relative level of responsibility of school staff in issues relating to curriculum and assessment (RESPCUR) is positively associated with the decrease in the hindrance in the learning climate among public schools with a high proportion of non-native language speakers. This finding implies that curricular responsibility is a potential mechanism to improve the learning climate.

Consistent with expectations, larger class size and the shortage of educational materials exacerbate learning climate hindrances are consistent across all our specifications. We also find similar patterns about parental involvement in its role in enhancing the learning climate among these schools who are likely to be facing social inclusion challenges. Parental inclusion in decision making is particularly vital in public schools with many students from disadvantaged homes, as well as schools’ identification and integration of community resources.

## Conclusions & limitations

In this study, we examined two critical questions. First, we analyzed the various roles parental involvement play in enhancing the school’s learning climate. Second, we identified the specific domains of parental involvement, which matter for schools facing issues of educational inclusivity. Statistically robust analyses covering a broad set of countries were performed. Several indicators of parental involvement were found to be strongly associated with principals’ perceptions of the school’s learning climate which are related to teacher behavior/management. Thus, this widens the opportunities and avenues for school leadership to improve the school learning climate. Our empirical findings are significant for public sector schools. They stand with the best potential to enhance the learning climate through activating plans of involving parents. Many public schools around the world are mandated to foster policies that encourage inclusive learning and this research hopes to clarify that enhancing inclusiveness is possible through parental involvement mechanisms.

Additionally, the paper’s conceptualization of the learning climate due to teacher behavior/management extends the conceptual unpacking of the learning climate in today’s learning contexts. Teacher roles are also continuously emphasized and redefined. From the simple conceptualization of the learning as the ‘quality and character of school life’ (Cohen et al., 2009) or the relational-social character of the schools with parents and the community (Maxwell et al., 2017), we show that the principal leadership continually possess a vital and holistic vantage point. The regression results from the multilevel framework do underscore the interdependencies between parental engagement and school leadership (Cohen et al., 2009; Thapa et al., 2013).

We spell out our work’s contribution to understanding the learning climate in two ways:

First is methodological unpacking. The specific forms of parental involvement presented are shown to be associated with improvements in the learning climate. The models were also extended to segments of schools with students coming from different social groups. Findings show that there are specific types of parental involvement that are actionable and enforceable. A welcoming environment for parental involvement matters considerably for public schools with SENs.

Moreover, we also find evidence that parental involvement is also not always positively related to learning climate outcomes. In one of the models, governmental laws requiring parental involvement is negatively associated with the learning climate. Such a relationship indicates that teachers and principals also face tensions involving parents. The models estimated have shown consistent results. However, emergent patterns should be further investigated at the sub-national or regional levels by other scholars in the field, particularly with the 2018 PISA round.

Second, we also revealed the critical components of school leadership domains in our analysis. The principal’s leadership in framing and communicating school goals and curricular development to the school are highly relevant in explaining educational inclusiveness. In practical terms, these include the principal’s use of student performance results, the congruency of teacher professional development activities with the teaching goals, teachers’ work habits, and the discussion of academic objectives with teachers during faculty meetings in the exercise school management (Schulz, 2003). Based on the robust empirical strategy this paper has employed, these construct(s) about principal leadership are generalizable to entire education systems. Policy implications can be directly drawn, most notably for practitioners.

### Limitations and future research

Research on the learning climate will undoubtedly be more relevant in the years ahead, cutting across a wide variety of themes of importance to policymakers, scholars, and practitioners. Tensions between teacher roles, principal leadership, and parental involvement need further attention. Our research certainly recognizes that our current research design has limitations, which certainly offers new work potential.

First, our empirical work draws only on cross-sectional data surveyed by the OECD in 2015. This situation raises two critical issues on the study of learning climate and parental involvement on our end. One, this certainly limits us to draw causal inferences and allows us at best to draw correlational and associations among the observable characteristics within the school. Further studies can consider experimental or quasi-experimental approaches regarding parental involvement.

Two, as the background literature shows, learning climate is both a complex and a dynamic character of schools. Hence, cross-sectional designs can only afford a stationary shot view within specific units of observation. Our measure of the learning climate is just one of the proxies of varieties of learning climate measurement. The learning climate attributable to student behavior also needs further attention. Nevertheless, it is operationally challenging to draw causal inferences across national education systems, and caution should be exercised whenever comparative studies are required. Future empirical work may also consider longitudinal designs, which brings on depth and changes over time. Yet, this work has shown many essential findings of how the learning climate can be actionable concerning parental involvement and school management, despite these constraints and limitations.

Lastly, in the light on the ongoing Covid-19 pandemic, the conceptualization of learning climate within online spaces shall also emerge as a critical research tradition. The tensions and knowledge gaps surrounding such systems and platforms’ inclusiveness will be essential research areas. These topics are inextricably linked with the practice of school leadership and management for the years ahead.

## Supplementary information

**Additional file 1.**

## Data Availability

All datasets, questionnaires, and codebooks are available from the OECD-PISA webpage, https://www.oecd.org/pisa/.

## Abbreviations

CFA: Confirmatory Factor Analyses
EFA: Exploratory Factor Analyses
HLM: Hierarchical Linear Modeling
ICC: Intraclass correlation coefficient
IRT: Item Response Theory
OECD: Organization for Economic Cooperation and Development
PISA: Program for International Student Assessment
SEN: Special Education Needs
RESPCUR: Index of responsibility of the school staff with issues relating to curriculum and assessment
RESPRES: Index of relative responsibility of staff in managing school resources
STANTEST1: Index of use of standardized tests indicating information for decision making
STANTEST2: Index of use of standardized tests indicating practices of comparison
LEADCOM: Index of the principal’s leadership in framing and communicating school goals and curricular development
LEADINST: Index of principals’ instructional leadership
LEADPD: Index of the principals’ promotion of instructional improvements and professional development among teachers
LEADTCH: Index of principals’ teacher participation
CLSIZE: Class size
STRATIO: The student-teacher ratio
SCHAUT: Overall index school autonomy
EDUSHORT: Index which captures the shortage of educational materials
STAFFSHORT: Index which captures the shortage of instructional staff
PROATCE: Index which captures the proportion of teachers in a school who are fully certified with teaching qualifications
SCHLTYPE: School type, indicating either private, public, or publicly funded private

## Footnotes

1 We conducted EFA using our earlier technique on these items, and we yielded two possible factors. However, the corresponding factor loadings of the items are quite low; altogether, their Cronbach alphas are also very low, as well as the values of average inter-item correlations. Proceeding with the CFA, our test shows that while the RMSEA and CFI indices are acceptable, but the TLI is quite low. Moreover, while performing such tests, we found valuable insights concerning data reduction and reduce the likelihood of multicollinearity. In several iterations, one item shifts its loading into either factor. For this purpose, we propose to integrate these parental involvement items individually.

2 For the purposes of brevity, construction of these variables is explained in step-by-step detail at the Additional file 1.

3 In this study, the SEN qualifier on the SC048 section of the PISA principal survey was used. Additionally, OECD has a present call for studies and long term research strategies “to seek ways to widen access of PISA for students with disabilities and other special education needs” https://www.oecd.org/pisa/pisaproducts/pisa-2024-call-for-tender-documents-for-bidders.htm.
