# Supplementary material for: School learning climate in the lens of parental involvement and school leadership: lessons for inclusiveness among public schools
Source: Smart Learn Environ. 2020 Sep 21;7(1):25. doi: 10.1186/s40561-020-00139-2 (PMC7504873; doi:10.1186/s40561-020-00139-2)
Supplement: Supplementary file 1 — Additional file 1. [file 40561_2020_139_MOESM1_ESM.docx]

**SUPPLEMENTARY MATERIALS: APPENDICES**

For the benefit of future PISA-related work on learning climate, we present step-by-step methodological details on how composite indicators/indices are constructed in the study.

1. **Methodological notes on constructing a composite indicator**
2. **Schools’ learning climate**

There are two possible factors which capture the learning climate within schools: (1) pertaining to student behavior (LEARNHIND) and (2) pertaining to management of teacher behavior (TEACHIND). We performed Bartlett's test for sphericity and have also computed for the Kaiser-Meyer-Olkin (KMO) measure of sampling adequacy. For both, we reject that the null hypothesis that the variables are not inter-correlated. Both measures of the learning climate, we obtained Cronbach’s alpha of above 0.8, implying a good measure of internal consistency. Graphically, we illustrate this in Figure 4 of this section.

The average inter-item correlations are 0.48 and 0.47 for both factors. However, the comparative fit index (CFI), Tucker-Lewis Index (TLI) and the root mean square error of approximation (RMSEA) do seem to suggest that a CFA model of the first factor (LEARNHIN) is not as suitable as expected because the RMSEA is at 0.230, well above the 0.08 ideal cut-off. The CFI and TLI indices are also below 0.95, though the standardized root-mean-square residual (RSMR) is only borderline acceptable at 0.078.

As a constructed index in our work, the compositeness of the LEARNHIND may raise issues when it comes to capturing the learning hindrance, even it is still highly correlated with the IRT-based measure developed by PISA. These summary statistics are shown in Table 4 of this section.

1. **Student assessment and evaluation.**

Principals were also asked about their usage of tools to monitor the practice of teachers in the school, i.e., whether they use tests or assessments of student achievement, teacher peer review, internal classroom observations, and/or classroom evaluations by persons external to the school (SC032).

Moreover, the constructed models included the frequency of deployment of student assessments. These are on mandatory and non-mandatory tests, teacher-developed tests, and teachers’ judgmental ratings. If respondents showed that there is more than one occurrence of these tests, principals were also asked to differentiate how are standardized, and teacher-developed tests are used (i.e., “to guide students’ learning, to inform parents about their child’s progress among other things” from the OECD 2015 Questionnaires.). In general, we find that the usage of standardized tests is more unidimensional across principals, as evidence by Cronbach’s alpha equal to 0.8829. The average inter-item correlation is 0.4072. Teacher developed tests have a reasonable internal consistency, with α equal to 0.7209 and inter-item correlation equal to 0.1901. These are in section SC035 of the survey. Using these preliminary indicators, we developed two measures based on how these tests are by the schools based on the EFA-CFA checks.

For standardized tests, two purposes generally emerged: one indicating information for decision making (i.e., “inform parents about their child’s progress, “to make decisions about students’ retention,” to make judgments about teachers’ effectiveness,” “to guide students' learning” and “ to award certificates to students.” The other dimension described practices of comparison, i.e., “compare the school to the district, state, or national performance,” “monitor the school’s progress from year to year,” and “to compare the school with other schools.”

The fit indices yielded a reasonable fit for the full sample. We label these two factors, STANTEST1 and STANTEST2, respectively, in our succeeding analyses. For teacher-developed tests, we did not find reasonable uni-dimensionality with regards to purpose. This finding is expected as there is high variability with teacher-developed tests. These vary within schools, too.


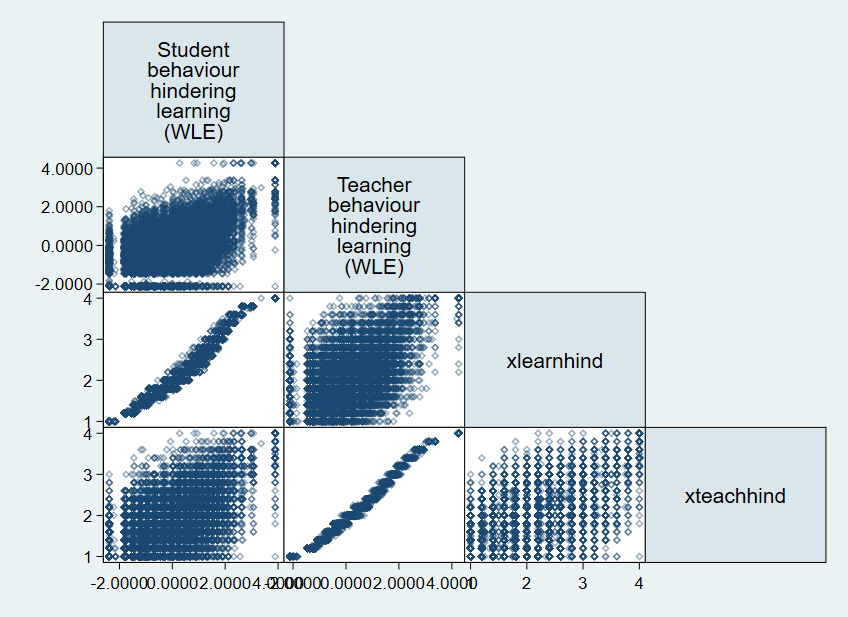


***Figure 4 Correlation between computed indices as proxies for learning climate, XLEARNHIND and XTEACHHIND, with the PISA composite indicators***

**Table 0. List of countries included with number of schools included. Note: simple tabulations of unweighted counts. Weighted number of responses are indicated in Table 4.**

| **Country** | **Freq** | **Percent** |  | **Country** | **Freq** | **Percent** |
| --- | --- | --- | --- | --- | --- | --- |
|  |  |  |  |  |  |  |
| Albania | 230 | 1.28 |  | Lithuania | 311 | 1.74 |
| Algeria | 161 | 0.9 |  | Luxembourg | 44 | 0.25 |
| Australia | 758 | 4.23 |  | Macao | 45 | 0.25 |
| Austria | 269 | 1.5 |  | Malta | 59 | 0.33 |
| Belgium | 288 | 1.61 |  | Mexico | 275 | 1.54 |
| Brazil | 841 | 4.7 |  | Moldova | 229 | 1.28 |
| Bulgaria | 180 | 1.01 |  | Montenegro | 64 | 0.36 |
| Canada | 759 | 4.24 |  | Netherlands | 187 | 1.04 |
| Chile | 227 | 1.27 |  | New Zealand | 183 | 1.02 |
| Chinese Taipei | 214 | 1.19 |  | Norway | 229 | 1.28 |
| Colombia | 372 | 2.08 |  | Peru | 281 | 1.57 |
| Costa Rica | 205 | 1.14 |  | Poland | 169 | 0.94 |
| Croatia | 160 | 0.89 |  | Portugal | 246 | 1.37 |
| Czech Republic | 344 | 1.92 |  | Qatar | 167 | 0.93 |
| Denmark | 333 | 1.86 |  | Romania | 182 | 1.02 |
| Dominican Republic | 194 | 1.08 |  | Russian Federation | 210 | 1.17 |
| Estonia | 206 | 1.15 |  | Singapore | 177 | 0.99 |
| Finland | 168 | 0.94 |  | Slovak Republic | 290 | 1.62 |
| France | 252 | 1.41 |  | Vietnam | 188 | 1.05 |
| Georgia | 262 | 1.46 |  | Slovenia | 333 | 1.86 |
| Germany | 256 | 1.43 |  | Spain | 201 | 1.12 |
| Greece | 211 | 1.18 |  | Sweden | 202 | 1.13 |
| Hong Kong | 138 | 0.77 |  | Switzerland | 227 | 1.27 |
| Hungary | 245 | 1.37 |  | Thailand | 273 | 1.52 |
| Iceland | 124 | 0.69 |  | Trinidad and Tobago | 149 | 0.83 |
| Indonesia | 236 | 1.32 |  | United Arab Emirates | 473 | 2.64 |
| Ireland | 167 | 0.93 |  | Tunisia | 165 | 0.92 |
| Israel | 173 | 0.97 |  | Turkey | 187 | 1.04 |
| Italy | 474 | 2.65 |  | FYROM | 106 | 0.59 |
| Japan | 198 | 1.11 |  | United Kingdom | 550 | 3.07 |
| Jordan | 250 | 1.4 |  | United States | 177 | 0.99 |
| Korea | 168 | 0.94 |  | Uruguay | 220 | 1.23 |
| Kosovo | 224 | 1.25 |  | B-S-J-G (China) | 268 | 1.5 |
| Lebanon | 270 | 1.51 |  | Spain (Regions) | 976 | 5.45 |
| Latvia | 250 | 1.4 |  | Argentina | 58 | 0.32 |
|  |  |  |  |  |  |  |
|  |  |  |  | Total | 17,908 | 100 |

**Table 1. Summary Statistics, Interitem correlations, and confirmatory factor statistics for school-level outcomes.**

| Item Code | Question | Obs | Mean | Std Dev | Interitem correlations and Cronbach’s alpha | CFA statistics |
| --- | --- | --- | --- | --- | --- | --- |
| SC061Q01TA | Student truancy | 16,319 | 2.28 | 0.85 | Ave. Inter-item correlation:  0.4839  Cronbach’s α:  0.8242 | RMSEA: 0.230***  CFI: 0.859  TLI: 0.717  SRMR: 0.078 |
| SC061Q02TA | Students skipping classes | 16,289 | 2.20 | 0.82 |  |  |
| SC061Q03TA | Students lacking respect for teachers | 16,278 | 2.02 | 0.76 |  |  |
| SC061Q04TA | Student use of alcohol or illegal drugs | 16,269 | 1.59 | 0.75 |  |  |
| SC061Q05TA | Students intimidating or bullying other students | 16,288 | 1.83 | 0.69 |  |  |
|  |  |  |  |  |  |  |
| SC061Q06TA | Teachers not meeting individual students’ needs | 16,283 | 1.96 | 0.76 | Ave. Inter-item correlation:  0.4685  Cronbach’s α:  0.8151 | RMSEA: 0.052  CFI: 0.991  TLI: 0.982  SRMR: 0.016 |
| SC061Q07TA | Teacher absenteeism | 16,320 | 1.82 | 0.78 |  |  |
| SC061Q08TA | Staff resisting change | 16,270 | 2.04 | 0.84 |  |  |
| SC061Q09TA | Teachers being too strict with students | 16,278 | 1.84 | 0.69 |  |  |
| SC061Q10TA | Teachers not being well prepared for classes | 16,284 | 1.82 | 0.74 |  |  |

Note: ***p<0.01. Computed from the 2015 principal survey data for which we have data. The first five variables capture the extent that student-related behavioral issues hinder learning; the next five captures the extent that teacher management/behavior issues hinder learning

**Table 2 Summary Statistics, Dimensions of Principal Leadership**

| Variable | Question | Obs | Mean | Std. Dev. | Min | Max |
| --- | --- | --- | --- | --- | --- | --- |
|  |  |  |  |  |  |  |
| SC009Q01TA | I use student performance results to develop the school’s educational goals. | 16,334 | 2.98 | 1.08 | 1 | 6 |
| SC009Q02TA | I make sure that the professional development activities of teachers are in accordance with the teaching goals of the school. | 16,310 | 3.31 | 1.25 | 1 | 6 |
| SC009Q03TA | I ensure that teachers work according to the school’s educational goals. | 16,302 | 3.98 | 1.31 | 1 | 6 |
| SC009Q04TA | I promote teaching practices based on recent educational research. | 16,267 | 3.38 | 1.39 | 1 | 6 |
| SC009Q05TA | I praise teachers whose students are actively participating in learning. | 16,276 | 4.06 | 1.35 | 1 | 6 |
| SC009Q06TA | When a teacher has problems in his/her classroom, I take the initiative to discuss matters. | 16,279 | 4.32 | 1.33 | 1 | 6 |
| SC009Q07TA | I draw teachers’ attention to the importance of students’ development of critical and social capacities. | 16,275 | 3.99 | 1.33 | 1 | 6 |
| SC009Q08TA | I pay attention to disruptive behavior in classrooms. | 16,287 | 4.87 | 1.23 | 1 | 6 |
| SC009Q09TA | I provide staff with opportunities to participate in school decision-making. | 16,261 | 4.24 | 1.22 | 1 | 6 |
| SC009Q10TA | I engage teachers to help build a school culture of continuous improvement. | 16,259 | 4.38 | 1.25 | 1 | 6 |
| SC009Q11TA | I ask teachers to participate in reviewing management practices. | 16,250 | 3.29 | 1.36 | 1 | 6 |
| SC009Q12TA | When a teacher brings up a classroom problem, we solve the problem together. | 16,252 | 4.62 | 1.25 | 1 | 6 |
| SC009Q13TA | I discuss the school’s academic goals with teachers at faculty meetings. | 16,306 | 3.70 | 1.10 | 1 | 6 |
|  |  |  |  |  |  |  |
| LEADCOM | SC009Q01TA SC009Q02TA SC009Q03TA SC009Q13TA | 16,355 | 0.18 | 0.98 | -4.83 | 3.00 |
| LEADINST | SC009Q04TA SC009Q05TA SC009Q07TA | 16,121 | 0.11 | 0.99 | -3.97 | 2.23 |
| LEADPD | SC009Q06TA SC009Q08TA SC009Q12TA | 16,117 | 0.17 | 1.05 | -3.81 | 1.81 |
| LEADTCH | SC009Q09TA SC009Q10TA SC009Q11TA | 16,092 | 0.09 | 1.04 | -3.86 | 2.40 |

Note: Median Cronbach’s alphas: Overall, 0.887; LEADCOM: 0.714; LEADINST: 0.722; LEADPD: 0.781; and LEADTCH, 0.780. Note: We arrived at an RMSEA of 0.078, CFI, and TLI levels of 0.938 and 0.917, respectively; the OECD has also computed scale reliabilities for these composite indicators for each country, and these have resulted in generally reasonable values. The variable LEADCOM has most of the number of Cronbach’s alpha below 0.70 (among 13 out of 37 countries), followed by LEADINST (12 countries), LEADPD (six countries), and LEADTCH (only 3). This pattern implies that the index of teacher participation in leadership is most consistent across most countries, while how the school’s goals and curricular development are framed and communicated vary the most. This an essential practical dimension of school management because principals across the world employ different managerial techniques in handling school operations.

**Table 6 Summary Statistics and Correlation Matrix**

**Table 4 Weighted mean values of school climate indicators as computed from the PISA 2015 dataset.**

| Country Identifier | Mean | Mean | Mean | Mean | Mean | *Mean* | Mean | Mean | Mean | Mean | Mean | *Mean* | Number of weighted responses |
| --- | --- | --- | --- | --- | --- | --- | --- | --- | --- | --- | --- | --- | --- |
|  | SC061Q01TA | SC061Q02TA | SC061Q03TA | SC061Q04TA | SC061Q05TA | ***xlearnhind*** | SC061Q06TA | SC061Q07TA | SC061Q08TA | SC061Q09TA | SC061Q10TA | ***xteachhind*** | n |
| Albania | 1.9 | 1.62 | 1.51 | 1.08 | 1.36 | *1.5* | 1.37 | 1.47 | 1.53 | 1.81 | 1.47 | *1.53* | 5000 |
| Algeria | 2.79 | 2.04 | 2.16 | 1.29 | 1.68 | *2* | 1.9 | 2.42 | 2.1 | 1.95 | 2.22 | *2.12* | 5000 |
| Australia | 2.09 | 1.97 | 2.02 | 1.71 | 2.05 | *1.97* | 2.28 | 1.91 | 2.22 | 1.72 | 1.93 | *2.01* | 5000 |
| Austria | 2.39 | 2.35 | 2.08 | 1.47 | 2.11 | *2.08* | 1.78 | 1.9 | 2.09 | 1.79 | 1.56 | *1.83* | 5000 |
| Belgium | 2.48 | 2.34 | 2.32 | 1.96 | 2.37 | *2.29* | 2.05 | 2.29 | 2.27 | 1.92 | 2.02 | *2.11* | 5000 |
| Brazil | 2.53 | 2.4 | 2.39 | 1.58 | 1.66 | *2.11* | 1.95 | 1.82 | 2.12 | 1.69 | 1.75 | *1.87* | 5000 |
| Bulgaria | 2.33 | 2.58 | 2.18 | 1.68 | 2.03 | *2.17* | 1.76 | 1.69 | 1.67 | 1.5 | 1.85 | *1.69* | 5000 |
| Canada | 2.47 | 2.25 | 1.97 | 1.97 | 1.91 | *2.11* | 2.02 | 1.66 | 2.09 | 1.74 | 1.71 | *1.85* | 5000 |
| Chile | 1.82 | 1.89 | 2.14 | 1.75 | 1.9 | *1.9* | 2.22 | 2.18 | 2.38 | 1.96 | 2.25 | *2.2* | 5000 |
| Chinese Taipei | 1.67 | 1.72 | 1.95 | 1.46 | 1.66 | *1.69* | 2.12 | 1.34 | 2.27 | 1.96 | 2.02 | *1.94* | 5000 |
| Colombia | 2.5 | 1.89 | 1.97 | 1.56 | 1.8 | *1.94* | 1.82 | 1.71 | 2.06 | 1.93 | 1.59 | *1.82* | 5000 |
| Costa Rica | 2.63 | 2.68 | 1.99 | 2.19 | 2.06 | *2.31* | 2.16 | 2.11 | 2.24 | 2.1 | 1.85 | *2.09* | 5000 |
| Croatia | 3.09 | 2.88 | 2.52 | 1.8 | 1.85 | *2.43* | 2.1 | 1.71 | 2.31 | 2 | 2.13 | *2.05* | 5000 |
| Czech Republic | 2.03 | 2.69 | 2.2 | 1.5 | 1.85 | *2.05* | 1.74 | 1.82 | 1.84 | 1.78 | 1.52 | *1.74* | 5000 |
| Denmark | 2.27 | 1.92 | 1.95 | 1.38 | 1.79 | *1.86* | 1.92 | 2.04 | 1.88 | 1.61 | 1.66 | *1.82* | 5000 |
| Dominican Republic | 2.15 | 2.05 | 2.1 | 1.26 | 1.91 | *1.89* | 1.97 | 1.53 | 1.85 | 1.94 | 1.71 | *1.8* | 5000 |
| Estonia | 2.36 | 2.34 | 1.94 | 1.34 | 1.93 | *1.98* | 1.95 | 1.58 | 1.89 | 1.72 | 1.59 | *1.75* | 5000 |
| Finland | 2.18 | 2.08 | 2.2 | 1.51 | 2.09 | *2.01* | 2.03 | 1.86 | 1.94 | 1.63 | 1.7 | *1.84* | 5000 |
| France | 2.41 | 2 | 2.01 | 1.71 | 1.96 | *2.02* | 2.03 | 1.91 | 2.31 | 1.99 | 2.01 | *2.05* | 5000 |
| Georgia | 2.29 | 2.04 | 1.61 | 1.23 | 1.29 | *1.73* | 1.51 | 1.69 | 1.46 | 1.32 | 1.73 | *1.56* | 5000 |
| Germany | 2.23 | 2.07 | 2.12 | 1.89 | 2.14 | *2.09* | 1.86 | 2.3 | 2.15 | 1.8 | 1.98 | *2.02* | 5000 |
| Greece | 2.09 | 1.91 | 1.84 | 1.32 | 1.63 | *1.76* | 1.49 | 1.51 | 1.65 | 1.75 | 1.37 | *1.55* | 5000 |
| Hong Kong | 1.75 | 1.6 | 2.06 | 1.26 | 1.79 | *1.69* | 2.26 | 1.8 | 2.28 | 2.02 | 2.01 | *2.07* | 5000 |
| Hungary | 1.9 | 1.84 | 2.05 | 1.41 | 1.61 | *1.76* | 1.74 | 1.42 | 1.53 | 1.61 | 1.31 | *1.52* | 5000 |
| Iceland | 1.78 | 1.78 | 1.91 | 1.35 | 1.84 | *1.73* | 2.07 | 1.75 | 1.89 | 1.51 | 1.81 | *1.81* | 5000 |
| Indonesia | 2.11 | 1.74 | 1.49 | 1.06 | 1.21 | *1.52* | 1.35 | 1.74 | 1.09 | 1.72 | 1.51 | *1.48* | 5000 |
| Ireland | 2.62 | 1.94 | 1.98 | 1.69 | 2.03 | *2.05* | 2 | 1.91 | 2.08 | 1.79 | 1.87 | *1.93* | 5000 |
| Israel | 2.57 | 2.31 | 2.08 | 1.41 | 1.44 | *1.96* | 2.01 | 2.31 | 1.86 | 1.81 | 1.89 | *1.97* | 5000 |
| Italy | 2.37 | 2.35 | 2.05 | 1.46 | 1.77 | *2* | 1.96 | 1.83 | 2.52 | 1.98 | 2.01 | *2.06* | 5000 |
| Japan | 1.94 | 1.79 | 2.07 | 1.27 | 1.73 | *1.76* | 2.14 | 1.45 | 2.17 | 2.11 | 2.13 | *2* | 5000 |
| Jordan | 2.6 | 2.12 | 2.37 | 1.39 | 1.9 | *2.08* | 2.05 | 2.22 | 2.2 | 2.02 | 2.1 | *2.12* | 5000 |
| Korea | 2.04 | 1.78 | 2.36 | 1.64 | 1.95 | *1.95* | 1.86 | 1.08 | 1.6 | 1.83 | 1.68 | *1.61* | 5000 |
| Kosovo | 2.29 | 1.87 | 1.84 | 1.32 | 1.61 | *1.78* | 1.79 | 1.86 | 1.91 | 2.05 | 1.93 | *1.91* | 5000 |
| Lebanon | 2.01 | 1.76 | 1.88 | 1.27 | 1.84 | *1.76* | 1.8 | 1.94 | 2.03 | 1.93 | 1.86 | *1.91* | 5000 |
| Latvia | 2.31 | 2.24 | 2.19 | 1.44 | 1.67 | *1.97* | 1.68 | 1.38 | 1.71 | 1.88 | 1.61 | *1.65* | 5000 |
| Lithuania | 2.12 | 1.94 | 1.93 | 1.36 | 1.92 | *1.85* | 1.67 | 1.08 | 1.58 | 1.41 | 1.56 | *1.46* | 5000 |
| Luxembourg | 2.39 | 2.09 | 2.07 | 1.82 | 1.84 | *2.04* | 1.98 | 1.86 | 2.16 | 1.8 | 1.82 | *1.92* | 5000 |
| Macao | 2.24 | 2.02 | 2.24 | 2 | 2.33 | *2.17* | 2.47 | 2.27 | 2.27 | 2.02 | 2.32 | *2.27* | 5000 |
| Malta | 1.63 | 1.53 | 2.14 | 1.28 | 2.07 | *1.73* | 2.19 | 1.72 | 2.11 | 1.84 | 1.74 | *1.92* | 5000 |
| Mexico | 2.44 | 2.16 | 1.82 | 1.58 | 1.77 | *1.95* | 1.82 | 1.68 | 1.79 | 1.97 | 1.62 | *1.78* | 5000 |
| Moldova | 2.65 | 2.28 | 2.11 | 1.43 | 1.99 | *2.1* | 1.63 | 1.56 | 2.04 | 1.78 | 1.92 | *1.79* | 5000 |
| Montenegro | 2.65 | 2.22 | 2.02 | 1.28 | 1.79 | *1.99* | 1.77 | 1.76 | 1.98 | 1.9 | 1.66 | *1.81* | 5000 |
| Netherlands | 2.18 | 2.3 | 2.18 | 2.02 | 2.32 | *2.2* | 2.61 | 2.33 | 2.38 | 2.18 | 2.37 | *2.37* | 5000 |
| New Zealand | 2.31 | 2.14 | 1.84 | 1.87 | 1.92 | *2.01* | 2.22 | 1.72 | 2.29 | 1.83 | 1.82 | *1.98* | 5000 |
| Norway | 1.85 | 1.87 | 2.2 | 1.38 | 2.05 | *1.87* | 2.45 | 2.27 | 2.26 | 1.88 | 2.08 | *2.19* | 5000 |
| Peru | 2.16 | 2.04 | 1.61 | 1.35 | 1.54 | *1.75* | 1.88 | 1.87 | 2.05 | 1.97 | 1.97 | *1.95* | 5000 |
| Poland | 2 | 2.38 | 1.82 | 1.39 | 1.61 | *1.84* | 1.56 | 1.49 | 1.64 | 1.45 | 1.46 | *1.52* | 5000 |
| Portugal | 2.22 | 2.38 | 2.13 | 1.55 | 1.69 | *2* | 2.02 | 1.83 | 2.31 | 1.66 | 1.72 | *1.91* | 5000 |
| Qatar | 1.59 | 1.94 | 1.74 | 1.1 | 1.43 | *1.56* | 1.58 | 1.7 | 1.61 | 1.55 | 1.5 | *1.59* | 5000 |
| Romania | 2.31 | 2.26 | 1.89 | 1.09 | 1.7 | *1.85* | 1.44 | 1.21 | 1.8 | 1.65 | 1.29 | *1.48* | 5000 |
| Russian Federation | 2.89 | 2.92 | 2.32 | 1.73 | 1.85 | *2.35* | 2.17 | 1.9 | 2.01 | 1.94 | 2.2 | *2.05* | 5000 |
| Singapore | 1.88 | 1.75 | 1.76 | 1.1 | 1.88 | *1.67* | 2.1 | 1.58 | 1.96 | 1.86 | 1.92 | *1.88* | 5000 |
| Slovak Republic | 2.04 | 2.66 | 2.09 | 1.3 | 1.68 | *1.95* | 1.6 | 1.32 | 1.69 | 1.86 | 1.48 | *1.59* | 5000 |
| Vietnam | 2.31 | 2.08 | 1.58 | 1.36 | 1.81 | *1.83* | 1.7 | 1.64 | 1.37 | 1.81 | 1.72 | *1.65* | 5000 |
| Slovenia | 2.35 | 2.45 | 2.15 | 1.62 | 1.8 | *2.08* | 1.86 | 2 | 2.02 | 1.68 | 1.73 | *1.86* | 5000 |
| Spain | 1.91 | 2 | 2.08 | 1.41 | 1.76 | *1.83* | 1.72 | 1.41 | 2.1 | 1.84 | 1.71 | *1.76* | 5000 |
| Sweden | 2.25 | 2.5 | 1.97 | 1.57 | 1.92 | *2.04* | 2.09 | 1.87 | 1.92 | 1.47 | 1.81 | *1.83* | 5000 |
| Switzerland | 1.96 | 1.97 | 1.92 | 1.89 | 1.92 | *1.93* | 1.87 | 1.61 | 2.1 | 1.65 | 1.68 | *1.78* | 5000 |
| Thailand | 1.97 | 1.88 | 1.8 | 1.51 | 1.57 | *1.75* | 1.43 | 1.32 | 1.39 | 1.82 | 1.58 | *1.51* | 5000 |
| Trinidad and Tobago | 2.67 | 2.54 | 2.73 | 2.05 | 2.45 | *2.49* | 2.58 | 2.65 | 2.48 | 1.93 | 2.37 | *2.4* | 5000 |
| United Arab Emirates | 2.16 | 1.92 | 1.81 | 1.22 | 1.64 | *1.75* | 1.91 | 2.02 | 1.86 | 1.89 | 1.7 | *1.88* | 5000 |
| Tunisia | 3.03 | 2.44 | 2.09 | 1.75 | 2.05 | *2.27* | 1.93 | 2.57 | 2.24 | 2.1 | 1.81 | *2.13* | 5000 |
| Turkey | 2.69 | 2.53 | 2.18 | 1.28 | 1.76 | *2.09* | 2.47 | 1.92 | 2.04 | 1.51 | 1.96 | *1.98* | 5000 |
| FYROM | 2.18 | 1.92 | 1.77 | 1.21 | 1.37 | *1.69* | 1.52 | 1.44 | 1.55 | 1.66 | 1.35 | *1.5* | 5000 |
| United Kingdom | 1.73 | 1.72 | 1.94 | 1.57 | 1.84 | *1.76* | 2.1 | 2.02 | 1.97 | 1.65 | 1.78 | *1.9* | 5000 |
| United States | 2.48 | 1.95 | 2.11 | 1.94 | 2.02 | *2.1* | 1.9 | 1.65 | 1.92 | 1.75 | 1.75 | *1.8* | 5000 |
| Uruguay | 2.38 | 2.15 | 1.92 | 1.6 | 1.76 | *1.96* | 1.94 | 2.56 | 2.3 | 1.74 | 2.04 | *2.12* | 5000 |
| B-S-J-G (China) | 2.31 | 2.32 | 2.42 | 2.27 | 2.41 | *2.35* | 2.59 | 2.2 | 2.59 | 2.19 | 2.6 | *2.44* | 5000 |
| Spain (Regions) | 2 | 2.1 | 2.06 | 1.46 | 1.74 | *1.87* | 1.7 | 1.43 | 2.13 | 1.87 | 1.77 | *1.78* | 4987 |
| Argentina (Ciudad Autonoma de Buenos) | 2.45 | 2.33 | 1.51 | 1.56 | 1.55 | *1.88* | 1.93 | 2.44 | 2.5 | 1.89 | 1.47 | *2.05* | 260 |
| **Total** | **2.24** | **2.12** | **2.02** | **1.51** | **1.83** | ***1.95*** | **1.92** | **1.79** | **1.98** | **1.81** | **1.81** | ***1.86*** | **345,247** |
